# Rare Effects of Quinine on the Skin

**Published:** 1886-11

**Authors:** J. E. Roach


### RARE EFFECTS OF QUININE ON THE SKIN.

*Editor of Daniel's Texas Medical Journal:*

I NOTICE in the September number of thhe *Courier Record* a report of a case of rare effects of quinine on the skin by Dr. C. H. Wilkinson, of Galveston, which reminds me of a case which I was called to see August 20th, 1886,—the two year old child of Mr. C. P. M.—and found the little fellow suffering from an attack of intermittent fever. I at once made the following ℞ for the child: Quinine sul grs. xx, B. C. Soda gr. xii, Syr Yerba Santa ℥ii.

M. S. Teaspoonful every hour and a half until four or five doses of the mixtures was administered; this was in the afternoon. August

21st, at 11 o'clock, A. M. I called again, and to my surprise the mother informed me the baby was broke out all over like it was taking measles, and further stated she could not believe it to be measles as it had not been exposed to the disease at all.

After carefully examining the eruption I informed her that the breaking out was the effects of the quinine. I ordered her to discontinue the medicine and I put it on the following

℞: Liq. Pot. arsen M xx.
Aq. Pura ℥ii.

M. S. Teaspoonful three times a day. The child did not have any more chills, and made a good recovery.

Since that time I have had occasion to prescribe quinine again; it produced the same erythematous eruption.

This is the first case of the kind I have ever met with. I report this case merely to add another link to the chain of evidence that dermatic cinchonism is no myth. J. E. ROACH, M. D.

